# Gene therapy approaches for equine osteoarthritis

**DOI:** 10.3389/fvets.2022.962898

**Published:** 2022-09-28

**Authors:** Parvathy Thampi, R. Jude Samulski, Joshua C. Grieger, Jennifer N. Phillips, C. Wayne McIlwraith, Laurie R. Goodrich

**Affiliations:** ^1^Orthopaedic Research Center, C. Wayne McIlwraith Translational Research Institute, College of Veterinary Medicine, Colorado State University, Fort Collins, CO, United States; ^2^Gene Therapy Center, University of North Carolina, Chapel Hill, NC, United States

**Keywords:** osteoarthiritis, gene therapy, cartilage, adeno-associated viral vectors, translational

## Abstract

With an intrinsically low ability for self-repair, articular cartilage injuries often progress to cartilage loss and joint degeneration resulting in osteoarthritis (OA). Osteoarthritis and the associated articular cartilage changes can be debilitating, resulting in lameness and functional disability both in human and equine patients. While articular cartilage damage plays a central role in the pathogenesis of OA, the contribution of other joint tissues to the pathogenesis of OA has increasingly been recognized thus prompting a whole organ approach for therapeutic strategies. Gene therapy methods have generated significant interest in OA therapy in recent years. These utilize viral or non-viral vectors to deliver therapeutic molecules directly into the joint space with the goal of reprogramming the cells' machinery to secrete high levels of the target protein at the site of injection. Several viral vector-based approaches have demonstrated successful gene transfer with persistent therapeutic levels of transgene expression in the equine joint. As an experimental model, horses represent the pathology of human OA more accurately compared to other animal models. The anatomical and biomechanical similarities between equine and human joints also allow for the use of similar imaging and diagnostic methods as used in humans. In addition, horses experience naturally occurring OA and undergo similar therapies as human patients and, therefore, are a clinically relevant patient population. Thus, further studies utilizing this equine model would not only help advance the field of human OA therapy but also benefit the clinical equine patients with naturally occurring joint disease. In this review, we discuss the advancements in gene therapeutic approaches for the treatment of OA with the horse as a relevant patient population as well as an effective and commonly utilized species as a translational model.

## Introduction

Osteoarthritis (OA) is a debilitating, painful and often chronic degenerative condition, negatively impacting a significant percentage of the human population. It is the most common form of arthritis affecting nearly 30 million Americans and causing an economic loss of $186 billion annually (1, 2). It is also a significant clinical problem in horses with OA-associated lameness being the predominant factor contributing to diminished athletic function, inability to race, and perform sport horse activities (3–5). A U.S Department of Agriculture survey performed in horses attributed 60% of lameness to be related to OA which translates to millions of horses being impacted by this performance-limiting musculoskeletal condition (6). OA has a considerable economic impact on the equine industry, with the annual direct and indirect costs amounting to over $1 billion per year in the United States (7, 8). There is no cure for OA and treatment primarily revolves around managing symptoms by systemic and local pharmacological therapies including analgesics and non-steroidal anti-inflammatory agents (NSAIDs) (9), surgical approaches such as microfracture and chondroplasty (10–13), and regenerative medicine strategies using blood derived (ACS, APS, and PRP) (14–17) or cell-based approaches (ACI/MACI) (18–22). However, these are only effective at providing short-term relief and do not alter disease progression nor do they completely restore cartilage structure and function (4, 23). Therefore, the restoration of articular cartilage remains an unmet clinical need.

One of the challenges to studying osteoarthritis is the difficulty in finding experimental subjects to accurately mimic the pathology of human OA. Laboratory animal models, although inexpensive and easy to use, do not truly represent most aspects of human OA due to their differences in cartilage thickness and small joint size (24, 25). Dogs are reasonably good animal models for studying human OA due to their similarity in joint anatomy (26, 27); however, their companion animal status and associated ethical challenges have precluded their widespread use as an animal model (26, 27). Caprine models are more commonly used to study human OA; however, limitations such as variability in cartilage thickness and defect volume leads to inconsistencies in drawing experimental conclusions (26, 28). Minipigs (a smaller version of traditional pigs) are another suitable animal model for human OA studies (29). They have comparable cartilage thickness allowing for the creation of partial thickness (cartilage-only) defects; however, handling and housing challenges have limited their active use as an animal model (26, 30, 31).

In this regard, horses have been shown to be an ideal model due to the similarities in structural and functional anatomy of the synovial joints. The overall cartilage thickness (1.75–2 mm), subchondral bone characteristics and joint anatomy are similar between horses and humans (30, 32). In particular, the carpal and metacarpal joints of the equine forelimb have comparable size, tissue structure and biomechanical loading to human joints. Moreover, as these equine joints are responsible for 60–65% of weight bearing, they are the most susceptible to post traumatic OA induced by athletic training and secondary trauma (4, 5). As an experimental model, the large size of the equine system also allows for the use of similar imaging and diagnostic modalities as used in humans unlike traditional small animal models. Moreover, horses are amenable to controlled exercise such as treadmilling, analysis of joint function (lameness and joint effusion scoring), examination of internal structures using imaging such as radiographic, CT, MRI analysis, and minimally invasive arthroscopy (4, 33–35). Synovial fluid analysis offers a relatively non-invasive measure of therapeutic drug levels after intra-articular administration. However, this is challenging in small animal models due to the small joint size and proportionally small synovial fluid volume. In this regard, the ease of harvesting large volumes of synovial fluid under sedation is a significant benefit of using the equine model in OA studies (36, 37). Furthermore, horses sustain naturally occurring OA, frequently undergo arthroscopic procedures and commonly have their joints aspirated and treated and, therefore, are a clinically relevant patient population, further justifying the use of horses for preclinical analyses since the results from those studies would benefit clinical equine patients with naturally occurring disease in parallel. Limitations of the equine model including cost, handling/housing, long time to maturity and ethical concerns must be taken into consideration with the use of this large animal model.

## Principles of gene therapy

OA is a degenerative joint disorder and as joints are discrete enclosed spaces, the effects tend to be largely localized to the joints. While an imbalance between cartilage degradation and new matrix synthesis leading to cartilage damage is considered central to the pathogenesis of OA, the involvement of an inflammatory component has now been well-recognized. Recent literature suggests that an innate immune response mediated by the joint components such as synovial membrane, joint capsule, subchondral bone and ligaments is responsible for initiating and sustaining an immune-mediated inflammation in the diseased joint (38–41). It is important to recognize that early inflammation, in response to joint injury is beneficial for the repair process. However, progressive damage to the joint and failed tissue repair results in activation of stress signaling pathways which initiate and perpetuate a low-grade chronic inflammation leading to clinical OA (42–45). Anti-inflammatory agents can help in inhibiting a chronic inflammatory response to protect the cartilage from further damage. In recent years, targeted approaches to retain the beneficial effects of acute inflammation and prevent the progression to low-grade, chronic inflammation have been adopted using inducible transcription factors which regulate several genes of the inflammatory cascade. An example for this approach is the use of specific inhibitors of Nuclear factor-κB (NF-κB), a central inflammatory mediator which responds to a large variety of inflammatory and immune receptors (46, 47). However, as NF-κB is also involved in normal immune responses and cell survival (48, 49), a global inhibition strategy is not ideal and methods to selectively manipulate NF-κB to achieve safe and effective anti-inflammatory effects need to be explored (47).

With OA having a targetable inflammatory component, the intra-articular route is particularly well-suited for the delivery of anti-inflammatory therapeutics into the joint. As this involves direct delivery of the drug into the joint space, it has the potential advantage of reducing off-target and systemic adverse effects (50, 51). Although this seems straightforward theoretically, achieving adequate and long-lasting concentration of the therapeutic agent using this method poses certain challenges. The synovial fluid which fills the joint space and acts as a lubricant and a nutrient source for chondrocytes, is a dialysate of blood plasma. The increased pressure from the intra articular injections prompts rapid turnover and clearing of the drug molecules from the joint space, often within a half-life of 4–5 h (50, 52, 53). Thus, it is difficult to maintain adequate levels of the delivered drug long-term in the synovial fluid using intra-articular delivery. Gene therapy approaches were developed in an effort to address these drawbacks (54).

Gene therapy methods were designed with the goal of delivering therapeutic molecules directly into the joint space using vectors such that the cells' own machinery is programmed to endogenously and continuously produce high levels of the target protein at the site of injection (36, 55). This can be achieved either by using a plasmid/vector encoding a target protein (*in vivo* gene therapy) or modifying cells outside the body to produce the target protein (transgene) which can be reintroduced into the body (*ex vivo* gene therapy). The therapeutic targets for gene therapy approaches are either anti-catabolic (anti-inflammatory), anabolic, or both. Anti-catabolic factors primarily act by halting the inflammation-mediated degradation of cartilage while anabolic factors are geared towards chondrocyte proliferation and new matrix synthesis. While this approach has clear advantages compared to the delivery of recombinant proteins, the practical application has certain challenges. Firstly, for efficient transduction, the host cell needs to be metabolically active. A significant percentage of the cells need to be successfully transduced to produce detectable levels of protein (53, 56). Although this is a potential caveat, it is important to point out the possibility that the therapeutic levels of the target protein necessary to alter a disease state might be much lower than those needed for detection by routine assays. Further, the vectors and the modified cells must have low immunogenicity to evade detection by the host immune system to allow for prolonged transgene expression in the joint or to allow for periodic redosing without adverse immune responses (57–59). The cytotoxicity and immunogenicity associated with viral vectors also raises important safety concerns with using direct viral delivery systems. Achieving lasting clinically relevant levels of the therapeutic agent is vital for the successful treatment of a chronic condition such as osteoarthritis (60).

## *Ex vivo* gene therapy

A common method of delivering target drugs to the joint is using *ex vivo* gene therapy approaches where the cells harvested from the joint are transduced in culture and then transplanted back into the joint. Under ideal conditions, the transduced cells become an intrinsic site of protein production. *Ex vivo* approaches are relatively safe as it allows for rigorous quality control of the modified cells before reintroduction into the body. There is extensive literature to demonstrate the use of *ex vivo* delivery approaches using various vectors derived from retrovirus (61, 62), foamy virus (63), and adenovirus (64, 65) to transduce a variety of cell types. A study where autologous synovial fibroblasts modified with a recombinant retroviral vector to overexpress IL-1Ra was the first to use an *ex vivo* approach in the field of cartilage repair (66). Since then, there have been several studies investigating the feasibility of using modified cells to overexpress therapeutic gene products in joint tissues (67, 68). *Ex vivo* approaches are well-suited for use in the equine model. Autologous cell therapy, where cells are isolated from joint tissues, expanded in culture and administered back into the joints, is commonly used in horses (18, 19) and therefore, they are a particularly amenable system to model *ex vivo* gene therapy methods. The first such studies in horses utilized allogenic chondrocytes which were adenovirally transduced to overexpress IGF-1(69) and BMP7 (70), both of which resulted in improved cartilage healing when transplanted into cartilage defects. A more recent study has reported improved healing and defect filling when autologous chondrocytes transduced with AAVIGF-1 were evaluated in chondral defects (71). However, *ex vivo* gene delivery methods are time and labor intensive and therefore not ideal for clinical application. Another major disadvantage is the rapidity with which intra-articularly injected cells are cleared from the joint, often within 1–2 weeks, thus affecting the long-term efficacy of the approach (72, 73).

### *In vivo* gene therapy

Owing to the limitations of *ex vivo* gene delivery, direct *in vivo* delivery approaches have been extensively explored in the field of gene therapy (36, 74–83). Viral vector-based systems utilize the natural tendency of viruses to efficiently penetrate and translocate their genetic material into the host cell as part of the disease process. To create a viral vector system, the viral genome responsible for virulence is replaced by the gene of interest along with their regulatory sequences. This ensures that the virus retains their infectivity while limiting their pathogenicity and the possibility of integrating with the host genome which could lead to insertional mutagenesis and cancer (84). Although there are different viral vectors that are appropriate, there are several safety and efficacy criteria that must be met before a viral vector can be successfully used in gene therapy. Important safety issues centered around the use of viral vectors include the probability of integration of the vector genome with the host cell and the degree of immunogenicity of the vector. An important reminder of the safety risks of viral gene therapy was the death of a participant in a gene therapy clinical trial due to an immune reaction to a systemically administered adenoviral vector (85). Another instance of serious adverse effects to viral vectors was the development of leukemia in several subjects successfully treated for X-linked severe combined immunodeficiency using retroviral vectors (86, 87).

Direct *in vivo* approaches have been utilized extensively in the equine OA model. The relative ease of intra-articular injections and synovial fluid analysis make it a beneficial animal model for direct viral mediated gene therapy. Several viral vectors have been investigated in the equine system for their safety, transduction efficiency and long-term transgene expression including adenovirus (36, 79, 80), lentivirus (77, 88, 89), and adeno-associated virus (56, 81, 82, 90, 91). Adenoviral mediated delivery of IGF-1 into normal equine joints was shown to result in elevated and persistent synovial fluid levels without any adverse effects (80). Further, AAV mediated delivery of IL-1Ra in an equine model demonstrated sustained therapeutic transgene levels for at least 8 months post-injection. In addition, efficienct transduction of *in situ* equine chondrocytes and synoviocytes was observed up to 4 months following AAV mediated intra-articular delivery of GFP (82). Consistent with these findings, studies conducted by others have demonstrated consistent and stable transduction of equine joint tissues using viral vectors up to 12 weeks (91) and 24 weeks post injection (56), which speaks to the suitability of the equine model for direct gene therapy methods.

#### Viral vectors

Several viral vectors have been explored for the intra-articular delivery of therapeutics for cartilage repair. Adenoviral vectors were tested extensively in early studies in the equine model (36, 55, 92). Adenoviral vectors generate significantly elevated transgene expression; however, they are unable to achieve sustained expression longer than 4 weeks. In addition, adenoviral vectors have high immunogenicity and can lead to adverse tissue reactions (4, 36, 55). Retroviral vectors are also able to achieve high levels of target protein in the tissues, but these vectors need actively dividing cells to achieve efficient transduction and are therefore, are not feasible for use with chondrocytes, a cell with low turnover rate (66). Other vectors include herpes viruses which are highly cytotoxic in joints (93, 94), and lentiviruses which have been utilized in equine *in vitro* models (88, 95) and small animal models (89, 96, 97). However, lentiviruses show a high tendency for host integration and potential mutational adverse effects.

A significant improvement in adenoviral gene therapy was the development of replication defective E1-deleted first-generation adenoviral vectors (FGAds) (98–102). However, deletion of the E1 replication gene left the majority of the viral genome intact resulting in leaky expression of viral genes consequently leading to destruction of the transduced cells, chronic cytotoxicity and transient transgene expression. Subsequent deletion of replication genes E2 (second generation) and E3 (third generation) has progressed to the generation of helper-dependent adenoviral vectors (HDAds, gutted, gutless, or high capacity) with all of the viral coding sequences removed. Due to the complete absence of a viral genome, HDAds are capable of long-term high-level transgene expression without acute cytotoxicity. In addition, the lack of a viral genome offers additional advantages such as large cloning capacity (~37 kb) and a limited risk of insertional mutagenesis (103–105).

**Figure 1:**
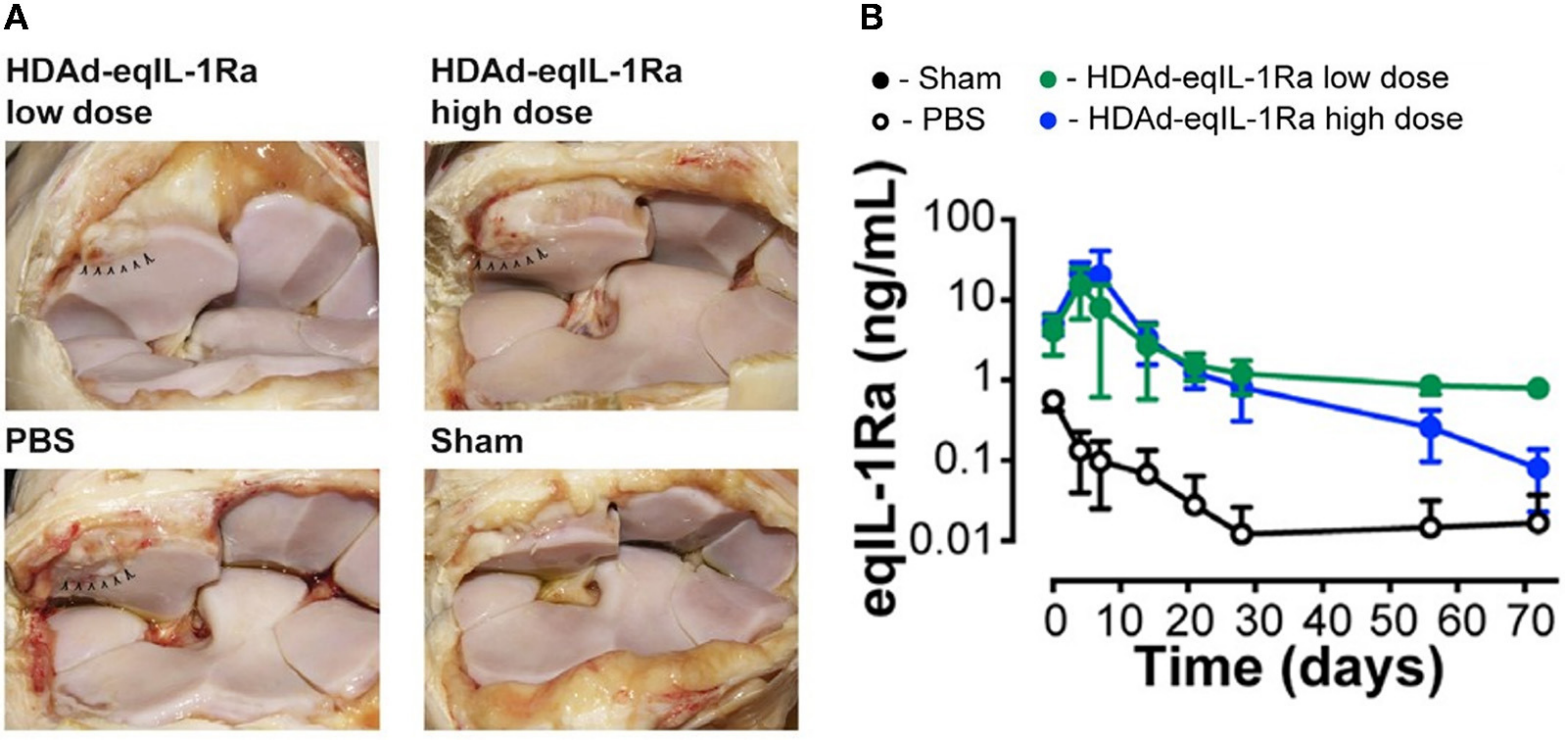
**(A)** Synovial membrane of OA joints injected with helper-dependent adenoviral vector encoding equine IL-1Ra (HDAd-eqIL-1Ra) at a low dose (2 × 10^11^ vp) or high dose (2 × 10^12^ vp) were comparable to sham-operated joints, whereas PBS-treated control joints appeared more hemorrhagic. Arrowheads indicate the site of osteochondral fragmentation. **(B)** Equine IL-1Ra levels in the synovial fluid peaked at 4 days after injection and declined to 1 ng/ml and 0.1 ng/ml in the low- and high-dose HDAd-eqIL-1Ra groups towards the end of the study. Values are represented as mean ± SEM. Reproduced with permission from Nixon et al., 2018 (114).

HDAds have been investigated extensively for liver-directed gene therapy in small animal models [reviewed in Brunetti-Pierri and Ng (104)]. These studies have demonstrated HDAds to provide long-term transgene expression with minimal cytotoxicity (106). The safety and efficacy of HDAd mediated therapy has also been explored in large animal models of liver disease including dogs (107–109) and non-human primates (110–112). In the non-human primate model, preferential delivery of HDAd vector into the liver resulted in transgene expression up to 7 years although with a gradual decline toward the end of the study (113). Further, HDAd vectors have been employed to deliver genes intra-articularly in mouse (114–116) and equine models of OA (114). In the horse OA model, treatment with HDAd-IL-1Ra resulted in a significant improvement in lameness scores, and cartilage and synovial membrane parameters suggesting an effective symptom and disease-modifying capacity as demonstrated in Figure 1. However, studies in large animal models have also revealed that HDAds show a dose-dependent acute cytotoxicity with the systemic route to administration (117). This cytopathic effect is not caused by viral gene expression, instead is an innate immune response triggered by the viral capsid. At high viral doses which is required for efficient transduction, acute cytotoxicity is observed, the level of which increases with dose. Therefore, strategies to block this host response or achieve efficient transduction at a sub-toxic dose need to be explored to overcome these challenges to clinical translation. In addition, the host inflammatory response against the viral capsid proteins is limited to high-dose systemic injections (104). This is not a limitation when using non-systemic routes of administration where HDAds can be directly delivered to isolated closed spaces thus minimizing the systemic cytotoxic effects. This is relevant in the context of OA therapy where HDAds can be administered intra-articularly (115) to reduce systemic cytopathic effects.

Adeno-associated virus (AAV) offers significant advantages over other viral vectors. It is a DNA parvovirus with a 4.68-kb genome composed of a linear single strand of DNA (118). AAV is unique in that it is naturally defective for replication and requires co-infection with a helper virus, most commonly adenovirus, to induce and support replication (119). This dependency on the helper virus for co-infection makes AAV non-pathogenic to humans (120, 121). Its recombinant form does not encode any viral proteins and thus has low chances of being recognized by the host immune system. Further, AAV vectors do not integrate into the genome of the host like lentiviruses. Various preclinical studies have demonstrated extended and successful transgene expression with AAV serotypes 2, 2.5, 5, and 8 (83, 122, 123). Unlike other viral vectors, AAV can transduce chondrocytes in addition to synoviocytes with a high level of transduction (83). Although the proportion of transduced cells are lower in cartilage than synovium, this is a major advantage considering that most viral vectors are unable to penetrate the extracellular matrix and efficiently transduce chondrocytes (53, 124–126). As chondrocytes are at the center of the OA pathogenesis, this is a major advantage of using AAV vectors for OA therapy. Importantly, AAV serotypes 2 and 2.5 use heparan sulfate, a vital ECM component of cartilage, as the primary binding receptor. This was found to be an important determinant of serotype dependent AAV transduction efficiency between cartilage explants and monolayer cultures which differ in their heparan sulfate content (127). The small particle size of the AAV vector allows it to enter and diffuse through the cartilage matrix to achieve effective transduction. However, this small capsid size also limits the transgene payload of AAV vectors to 4,100–4,900 bp, and this poses a problem for genes with large coding sequences, such as cystic fibrosis transmembrane conductance regulator gene (128, 129).

**Figure 2:**
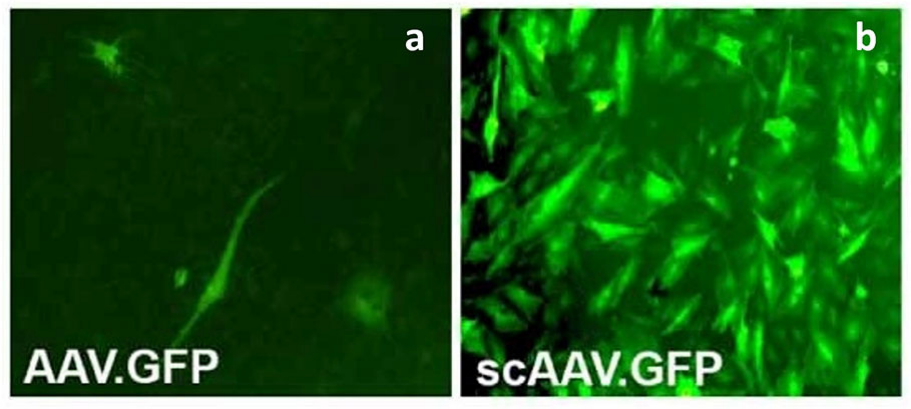
Primary articular fibroblasts from rabbits transduced with the same dose of conventional AAV encoding GFP (AAVGFP) **(a)** or double-stranded, self-complementary AAV encoding GFP (scAAVGFP) **(b)**. scAAV vectors produced a ~25-fold greater transduction compared to the conventional AAV vector. Reproduced with permission from Kay et al. (136).

AAV vector is composed of a single stranded DNA (ssDNA) genome, and the synthesis of the complementary strand occurs using the host's cellular replication factors by virtue of a palindromic terminal repeat (TR) structure which serves as a primer for synthesis of the complementary DNA strand (130–132). Therefore, transduction efficiency and onset of transgene expression is dependent on the conversion from the single to double strand DNA by the host cell and this is a major limiting factor of AAV vectors. This disadvantage has been overcome by the development of self-complimentary AAV vectors (scAAV) which are designed to self-generate both the + and –strand viral genomes without depending on the host cell (133, 134). scAAV vectors are composed of two halves of the ssDNA packaged separately such that they fold and base pair to form the dsDNA of half the length. While this reduces the average packaging capacity of scAAV vectors (~2.2 kb) to half of that of AAVs, this eliminates the need for host cell-dependent DNA synthesis which translates into higher efficiency and faster onset of transgene expression (135). scAAV vectors have demonstrated ~25-fold higher transduction efficiencies compared to the conventional AAV vectors as demonstrated in Figure 2 (136) and faster onset of protein production (81, 82, 133, 134, 136, 137). As packaging size is even more restricted in scAAVs compared to AAVs, optimization of transcriptional and post-transcriptional regulatory elements, as well as codon optimization, is vital to achieving high levels of transduction (82). The field of gene therapy has the potential to advance significantly with the recent development of CRISPR/Cas9 technology. In fact, a recent study demonstrated the feasibility of this approach using an adeno-associated virus, which expressed CRISPR/Cas9 components to target multiple genes simultaneously in an induced OA mouse model (138).

#### Non-viral vectors

Viral vector-based gene therapy offers the advantage of high transduction efficiency, however, it has not garnered unanimous acceptance due to the drawbacks of immunogenicity and cytotoxicity. In this context, non-viral vectors have been actively explored as an alternative. Non-viral vectors include naked-DNA and liposomes both of which comprise a plasmid, a circular closed DNA strand. The transgene is inserted into the plasmid directly followed by delivery into the cells. Non-viral gene delivery systems can be categorized into physical methods such as electroporation, sonoporation, photoporation, hydroporation, and magnetofection or chemical carriers such as inorganic particles and synthetic/biodegradables (139, 140). Compared to viral vectors, plasmids are relatively safe as there is no risk of integrating with the host genome which also allows for potential re-dosing with low immunogenicity. In addition, they are easier and cheaper to manufacture and have a longer shelf life. Owing to these advantages, plasmids have been used extensively in non-viral gene therapy approaches (141–143). A landmark study in the field of OA therapy involved the use of a plasmid to deliver a long-acting human interleukin-10 variant into the joints of companion dogs with naturally occurring osteoarthritis (144). This resulted in an improvement in pain measurements without any adverse effects in the double blinded placebo-controlled study. However, a major challenge with the existing non-viral methodologies is their inability to match the efficiency of viral vector-based systems.

### Challenges in gene therapy

The field of viral vector-based gene therapy has expanded significantly in recent years which translates to over 2000 clinical trials initiated since the 1990s and a drastic rise in commercial initiatives (145). However, this acceleration in the field has been accompanied by challenges associated with viral vector manufacturing capacity, vector characterization and increased regulatory scrutiny (146). Designing and manufacturing viral vectors successfully and consistently is expensive, and requires experienced scientists and high quality control (QC). These challenges have limited the widespread clinical application of gene therapy approaches (147). Viral vector manufacturing involves a variety of approaches, typically using mammalian cells in adherent or suspension systems. However, these systems are challenging to scale up due to the increased supply costs, processing time and batch to batch variation (148, 149). An alternative to overcome these limitations is the use of larger single-use culture systems and bioreactors which is increasingly being adopted in the field (150–154). Another source of variation in vector manufacturing arises from the use of transfection-based methods to generate vectors. Efficient transfection is highly dependent on the appropriate combination of transfection reagents, pH and plasmid DNA, and is highly susceptible to batch-to-batch variation. When scaling up for clinical manufacturing, this poses a significant barrier to producing consistent results with low lot-to-lot variability (155–158). Moreover, the transition of gene therapy approaches from pre-clinical animal studies to human clinical studies has prompted extensive characterization and QC testing of recombinant viral vectors to ensure batch-to-batch consistency. However, one of the limitations to characterizing and QC testing of viral vectors is their inherent degree of complexity. AAV, one of the smallest and simplest of recombinant viral vectors is significantly more complex than the most complex recombinant protein. Retroviral vectors are more complex with a double-stranded genome and a lipid bilayer encapsulating the capsid (146, 159). Thus, methods for characterizing viral vectors need to be customized for each different viral vector and each serotype.

Cell-based (*ex vivo*) gene therapy offers a different set of problems such as high sensitivity to environmental factors and intrinsic biological variability. Unlike conventional therapeutics, *ex vivo* gene therapy products are composed of live cells from the starting material to the final product. As the cells cannot be filtered or sterilized at the end of culture before use in the patient, the entire manufacturing process must be carefully designed and enforced with excellent QC strategies in place to ensure the safety and efficacy of the final product (160–162). The existing manufacturing systems for gene therapy are largely manual involving planar culture systems, which are difficult to scale-up and are riddled with batch-to-batch variability due to human error (163, 164). Cell based gene therapies typically use the patient's own cells as starting material which is also a source of variation owing to the inherent biological differences between donors. This variability can be considerably reduced when using allogenic or “off-the-shelf” therapies where cells from one individual can be used for multiple treatments. In contrast, autologous cell-based therapies, where cells taken from a patient are reintroduced into the same patient, present additional manufacturing challenges to control for the biological variation in the input material and inconsistent storage and preservation methods across samples (164–167). To add to these challenges, commercial gene therapy products are tightly regulated and are required to be produced in accordance with good manufacturing practice (GMP). However, this becomes challenging in the field of patient-specific (autologous) therapies where the cells are often sourced from a diseased joint and therefore, may not meet the required GMP standard (163, 168). In this context, gene therapy manufacturing processes would benefit from a more adaptive strategy that accounts for the inherent biological variation in the input material, differences in product quality and the complexity of viral systems. Technological improvements in viral manufacturing in future years is also likely to help in overcoming obstacles in large scale vector manufacturing and facilitating easier clinical translation.

## Horse as a translational OA model

### Pre-clinical studies using viral vectors

**Figure 3:**
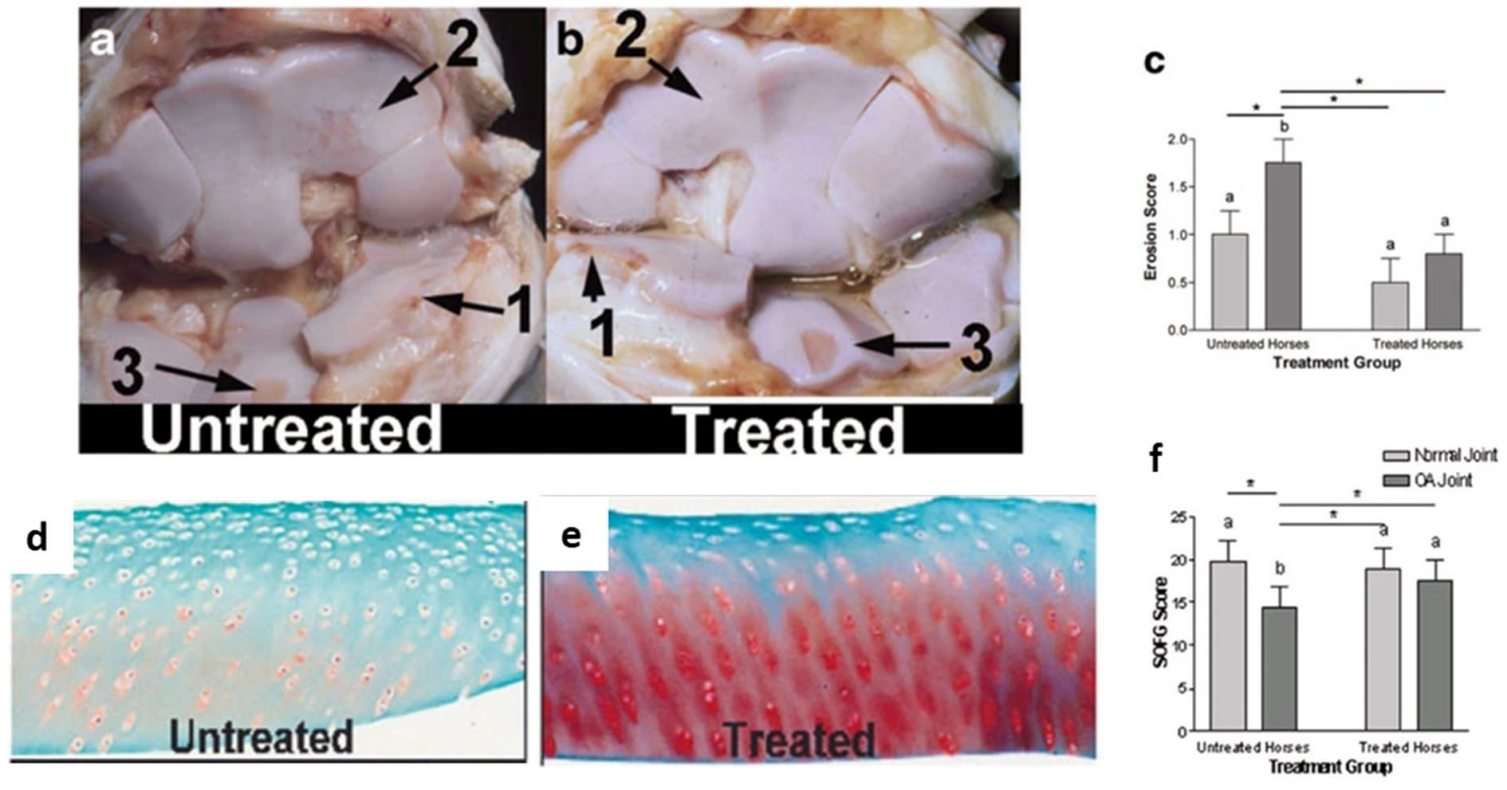
Effect of osteoarthritis and gene transfer on cartilage erosion. Photographs of the intercarpal joint illustrating extensive full-thickness articular cartilage erosions in OA joints of both untreated **(a)** and adenoviral vector encoding equine IL-1Ra (Ad-EqIL-1Ra) **(b)** treated horses. Erosions are evident in the untreated joint especially in areas of the third carpal bone (2) not adjacent to the osteochondral fragment (1). (3) shows the area of aseptic harvest of cartilage from the intermediate carpal bone. **(c)** presents cartilage erosion scores by treatment group. Sections of articular cartilage stained with SOFG demonstrating little or no stain uptake in the OA joint of an untreated horse **(d)** vs. moderate stain uptake from an OA joint with Ad-EqIL-1ra treatment **(e)**. Plot of SOFG scores by treatment group **(f)**. Different letters associated with bars indicate a statistical difference (*P* < 0.05) between bars. Lines with an asterisk (*) linking treatment groups indicate a statistical difference between treatment groups. Reproduced with permission from Frisbie et al. (36).

As illustrated in previous sections, horses offer an ideal model system for human OA. Numerous studies have demonstrated the advantages of viral gene delivery for the treatment of osteoarthritis in animal models. The purpose of this review article is not to provide a comprehensive review of all of these studies. Instead, examples of particular significance or interest are highlighted. Preclinical gene therapy studies in horses were performed by our group in 2002 with a first-generation adenoviral mediated delivery of Interleukin-1 receptor antagonist (IL-1Ra) into healthy equine joints. The study was successful in demonstrating a dose-dependent increase in IL-1Ra levels in synovial fluid, however, an acute leukocytosis was observed in the synovial fluid at the highest concentration tested (5 × 10^11^ viral particles) (36). Further, the efficacy of IL-1Ra to ameliorate the symptoms of OA was investigated in an induced equine OA model using an osteochondral chip fragment created in the intercarpal joint. Viral delivery of IL-1Ra resulted in increased intra-articular expression of IL-1Ra for ~28 days with a peak at 7 days. Moreover, the elevated IL-1Ra expression reduced joint pain and had a protective effect on the joint tissues over the course of the 90 day study as summarized in Figure 3 (169). While these were hallmark studies illustrating the success of viral mediated gene delivery into large animal joints, these studies used first-generation adenovirus vectors. An improvement in the field was the discovery of single-stranded adeno-associated viruses (AAVs) and subsequently of scAAV vectors generated using half-genome sized vector plasmids, or those containing a mutation in one of the terminal resolution sequences of the AAV ITRs which demonstrated greater transduction efficiencies and prolonged transgene expression compared to AAVs (81, 82, 133–135).

**Figure 4:**
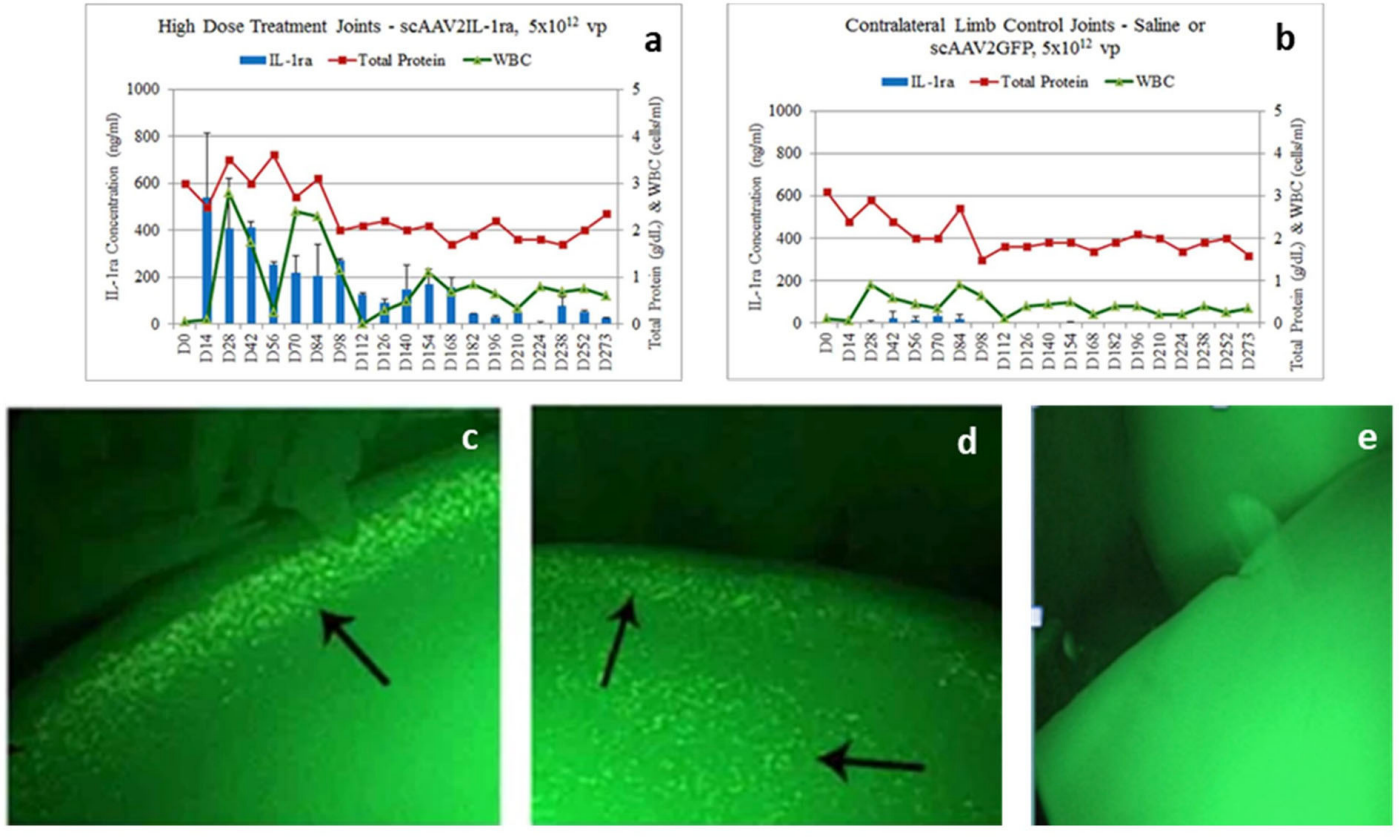
**(a,b)** IL-1Ra levels (blue bars), total protein levels (red line) and white blood cell (WBC) levels (green line) in the joints of horses in a scAAVIL-1Ra dosing trial. IL-1Ra levels remain elevated in joints injected with the highest dose of scAAVIL-1Ra **(a)** compared to the control joints (saline or scAAVGFP) **(b)**. Transduced joints produced very high levels of IL-1Ra (over 100 ng/ml) for over 168 days after which, levels of IL-1Ra continued to be produced between 50–100 ng/ml out to 273 days. WBC counts did not rise above normal (1,000 cells/ml) for joints injected with scAAVIL-1Ra and saline or scAAVGFP. **(c–e)** Arthroscopic image of *in situ* vector encoded-GFP transduced chondrocytes (arrows in left and middle images) from joints injected with 5 × 10^12^ vg of scAAVGFP (left) or saline (right image). The arthroscopic images were taken 4 months following intra-articular injection of scAAVGFP suggesting chondrocytes have been stably transduced to produce protein. The arrows point to fluorescent chondrocytes on the edge **(c)** or edge and surface of cartilage **(d)**. Some green autofluorescence (of the cartilage) surrounds the cells and can be seen in the saline injected joint **(e)**. scAAV, self-complementary AAV. Reproduced with permission from Goodrich et al. (81).

**Figure 5:**
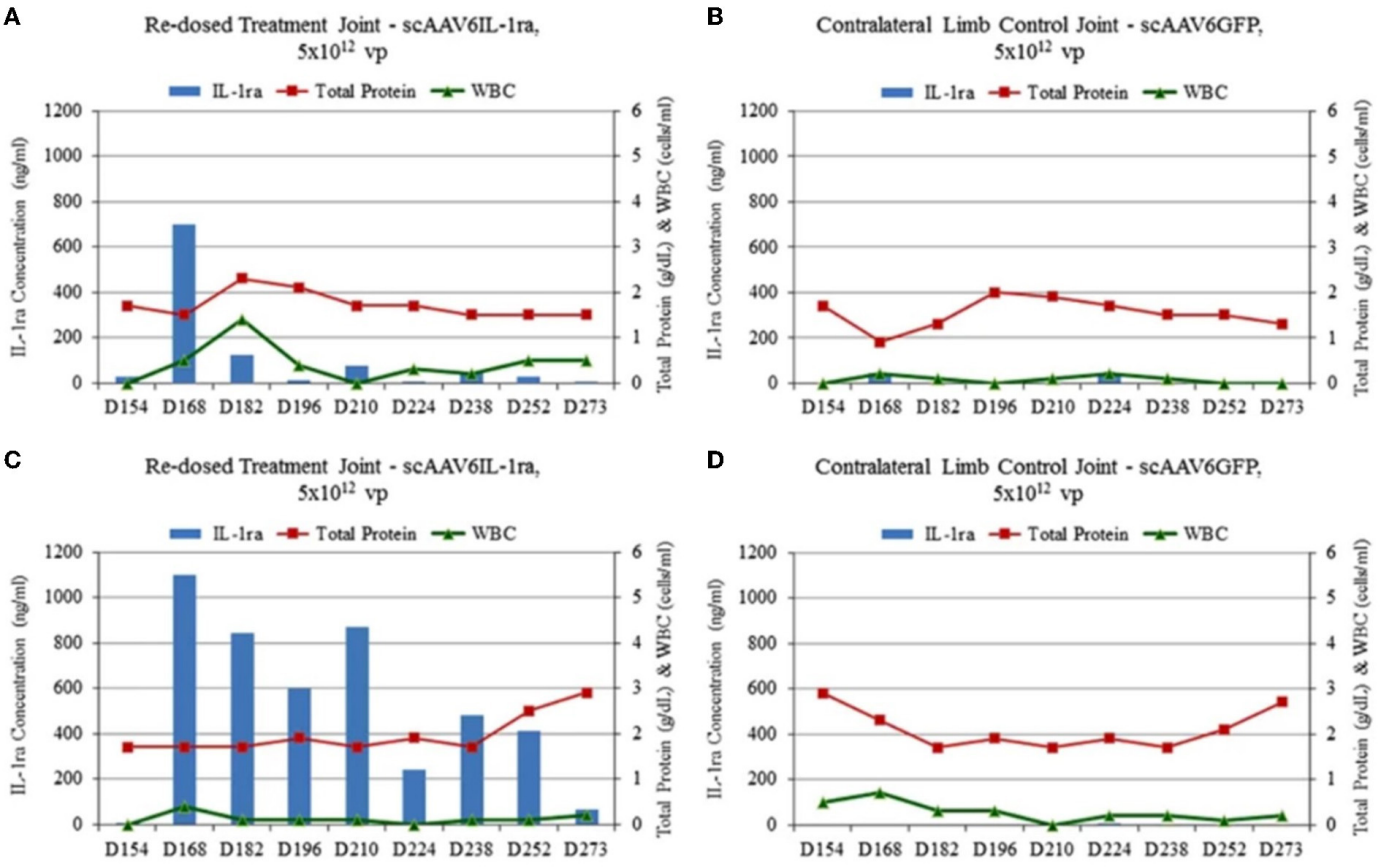
**(A–D)** IL-1Ra (ng/ml), total protein (g/dl) and WBC (cells/ml) levels in low dosed and control limb synovial fluid samples after redosing at Day 154. One horse **(A)** whose joint was re-injected had IL-1Ra level sharply increase to 675 ng/ml and decline rapidly while the other horse **(C)** that was redosed showed elevated IL-1Ra levels which remained elevated until Day 273 compared to the control joints **(B,D)**. Reproduced with permission from Goodrich et al., 2015 (81).

scAAV vector-based gene therapy approaches were successfully tried in laboratory animals (136, 170) before being tested in normal equine joints (56, 81, 82, 171, 172) and in an induced OA model (91). Goodrich et al. were the first to demonstrate *via* a novel fluorescent arthroscopic imaging system that *in situ* chondrocytes (in addition to synoviocytes) can be efficiently transduced with intra articular injection of scAAV GFP in equine joints (82). Watson et al. used scAAV containing the cDNAs for human IL-1Ra (scAAVhIL-1Ra) and green fluorescent protein (scAAVGFP) to transduce both equine and human synovial fibroblasts in culture. Of the AAV serotypes tested, AAV1, 2 and 5 were able to transduce both cell types at high efficiency with the equine cells showing a 10-fold higher viral uptake compared to the human cells. Further, delivery of these scAAV containing human IL-1Ra into the joints of equine forelimbs revealed biologically relevant levels of transgene expression mainly in the synovial cells and weakly in the articular chondrocytes. However, transgene expression steadily declined over a period of 5 weeks potentially due to detection and clearing of cells expressing the xenogenic human IL-1Ra protein by the host immune system (172). The human cDNA was later replaced with the homologous equine IL-1Ra, subsequent codon optimization, and optimizing the promoter for joint tissues resulting in a scAAV equine IL-1Ra vector which induced significant and therapeutic protein production in equine joints (82). This was followed by several dosing studies to identify dosing regimens to achieve prolonged and therapeutic levels of transgene expression (56, 81). In our study (81), scAAVIL-1ra, scAAV2GFP or saline at a dose ranging from 5 × 10^10^ to 5 × 10^12^ viral particles were delivered into the middle carpal space of six healthy horses. The dose of 5 × 10^12^ achieved therapeutic levels of IL-1Ra for at least 8 months following injection without any adverse effects (Figure 4). Moreover, re-dosing of the low dose groups with an alternate serotype demonstrated a rescue of IL-1Ra expression. Interestingly, one horse that was redosed with a scAAV6 IL-1Ra demonstrated a rescue of IL-1Ra levels at 2 weeks post injection but a rapid drop at 4 weeks suggestive of an immune response (Figure 5) while the other horse also redosed with the same serotype showed high levels of IL-1Ra which was sustained for over 100 days followed by a gradual decline. The results of this study suggest the ability to re-dose a patient with an alternate serotype and rescue IL-1Ra expression. Chondrocytes could be efficiently transduced using the dosing protocol tested in this study although the response to repeated dosing needs to be investigated further.

**Figure 6:**
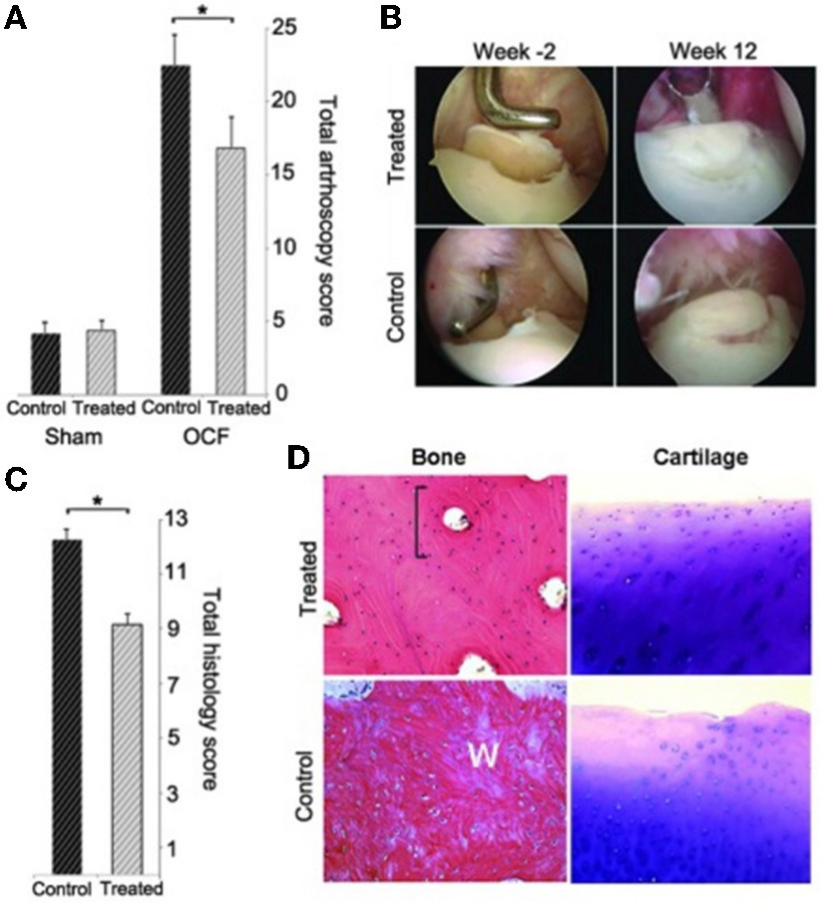
Changes in tissue pathology associated with scAAV.eqIL-1Ra treatment in an equine model of OA **(A)** showing a significant decrease in total arthroscopy scores for treated joints compared to the control joints **(B)** Representative arthroscopic images of the osteochondral lesions in treated and control horses at the time of OA induction (week -2) and at the endpoint (week 12) **(C)** showing a significant decrease in total histologic scores for treated joints compared to controls consistent with the microscopic appearance of bone repair tissue (H&E) and cartilage (toluidine blue) in treated and control joints **(D)** Reproduced with permission from R. S. Watson Levings et. al, 2018 (91). An asterisk (*) linking treatment groups indicate a statistical difference (*p* < 0.05) between treatment groups.

Similar patterns were observed in a 6-month study done by Watson Levings et al. (56). The viral dose of 5 × 10^12^ viral particles was found to result in stable therapeutic levels of IL-1Ra (over 35 ng/ml) with no adverse effects up to 24 weeks after intra-articular administration in healthy equine joints. In a related study, the same group of investigators tracked the expression of IL-1Ra in healthy vs. diseased joints. Interestingly, the vector activity appeared to be significantly higher in the joints with OA pathologies compared to healthy joints as demonstrated by a concentration of GFP fluorescence in the areas of articular cartilage with distinct damage. The investigators postulate that the increase in transgene expression could be a result of proliferation and increased metabolism of the diseased chondrocytes as well as stress induced activation of the cytomegalovirus (CMV) promoter which is normally activated by stress-activated protein kinases (173–175). These observations present a unique opportunity to target transgene expression to specific diseased regions of the joint and direct site-specific repair. Further, in a 12-week study, the same dose of 5 × 10^12^ of scAAV.eqIL-1Ra administered to joints of an induced equine OA model resulted in significantly elevated IL-1Ra expression levels in synovial fluid with a significant functional outcome of reduction in lameness, inflammatory responses and a chondroprotective effect at the site of injury (Figure 6) (91). While no adverse effects were observed in response to the viral dose tested in healthy horses (56, 81) and in a 12-week study in an induced OA model (91) summarized above, it is unclear how a diseased joint will behave long-term to this dose.

Gene therapy strategies in the realm of OA therapy can be broadly grouped into two–one aimed at delivering anti-catabolic gene products which halt the activity of inflammatory cytokines responsible for inflammation and breakdown of the cartilage ECM and the other directed toward delivering anabolic products that stimulate chondrocytes to proliferate and increase new matrix synthesis thus promoting cartilage regeneration.

Of the several anti-inflammatory factors tested for their efficacy in OA therapy, IL-1Ra is by far the most extensively investigated (36, 81, 93, 114, 176–179). IL-1Ra is the natural inhibitor of IL-1β and competitively inhibits IL-1β by binding to the surface receptors thereby preventing the cellular effects of IL-1β. As a potent mediator of the inflammation, IL-1β is responsible for the production of the major effectors of the inflammatory cascade including cyclooxygenases I and II, nitric oxide, phospholipase A2, prostaglandin E2, reactive oxygen species as well as inflammatory cytokines and chemokines which trigger degenerative changes in the cartilage matrix (180, 181). IL-1Ra, being a small protein [with a cDNA 1.6 kb in length], is ideal for gene therapy approaches using scAAV vectors which have packaging size limitations of ~2.5 kb (136, 182). IL-1Ra is being used clinically as a recombinant protein, Anakinra/Kineret^®^ in human and animal patients (183). This is administered as a daily subcutaneous injection, therefore the risk of toxic levels intra-articularly is low however, it does pose systemic side effects such as local reaction at the injection site and upper respiratory tract infections (184–186).

Insulin-like growth factor-1 (IGF-1) is a critical anabolic growth factor for maintaining cartilage health and integrity and for this reason, IGF-1 offers another useful target in OA therapy as a chondroprotective factor. *In vitro* studies provide strong support for the use of IGF-1 in promoting cartilage repair. IGF-1 has been shown to increase the metabolism and proliferation of chondrocytes in culture in several studies (187–189). The effect of IGF-1 was tested in an antigen-induced arthritis rabbit model using adenoviral-mediated delivery. Although IGF-1 levels were elevated in the joints which resulted in enhanced proteoglycan synthesis, this did not translate into a significant chondroprotective effect against OA pathology (190). IL-1Ra and IGF-1 have also been used in combination with the goal of simultaneously blocking cartilage breakdown and effecting cartilage regeneration, respectively (187). Synovial fibroblasts transduced with both IL-1Ra and IGF-1 were cocultured with normal equine articular cartilage and cartilage damaged by exposure to IL-1. The transgene expression from the synovial fibroblasts was able to increase matrix synthesis in normal cartilage as well as partially reverse the IL-1 induced cartilage matrix depletion in the damaged cartilage. Among large animal models, Goodrich et. al demonstrated that direct adenoviral mediated delivery of IGF-1 to the synovium of healthy equine joints was able to provide sustained high levels of IGF-I in the synovial fluid with minimal detrimental effects (80). Further, the chondroprotective effect of IGF-I was illustrated in an equine cartilage repair model (67). Chondrocytes genetically modified by an adenovirus vector encoding equine IGF-1 (AdIGF-1) administered into surgically induced cartilage defects resulted in improved repair of the cartilage defect with increased defect filling and elevated type II collagen expression compared to control defects.

Although IL-1Ra and IGF-1 have been the focus of OA gene therapy systems, significant advances in the field of viral vector design and generation opens the door for other potential gene targets known to play a role in cartilage development and regeneration. In fact, several of these have been investigated in *in vitro* studies and laboratory animal studies. SRY-Box Transcription Factor 9 (SOX9), a chondrocyte-specific transcription factor was tested in human and rabbit cell lines as well as in a rabbit *in vivo* model for degenerative disc disease using recombinant adenoviruses (191) and AAVs (192). Both studies demonstrated successful upregulation in SOX9 and Type 2 collagen levels and an associated protective effect on the architecture of the nucleus pulposus *in vivo*. Similar observations were made in another study investigating the effect of a recombinant adeno-associated viral (rAAV) vector mediated delivery of SOX9 in modulating the osteoarthritic phenotype of chondrocytes in a three-dimensional *in vitro* model (193). SOX9 has also been investigated in combinatorial gene delivery approaches in the field of cartilage regeneration (194).

Interleukin 10 (IL-10), an anti-inflammatory cytokine, which downregulates proinflammatory cytokines and its receptors, is another molecule that the gene therapy field has targeted. Over expression of this protein has been investigated in arthritis (195–200) and neural disorders (201–203). Human IL-10 gene carrying plasmids were designed by Watkins et. al. to test the safety and efficacy of IL-10 in a 6-month study in dogs. The therapy was well-tolerated without any adverse effects in the toxicology study. Subsequent testing in a translational model of OA in companion (pet) dogs with naturally occurring OA showed a significant improvement in pain measurements based on clinical assessments without any side-effects (144). Direct viral mediated delivery of IL-10 has also been examined in both *in vitro* and *in vivo* studies. Transduction of equine chondrocytes with AAV5 overexpressing IL-10 was able to mitigate the IL-1β mediated pro-inflammatory cascade in pellet cultures with a reduction in IL-1β and Prostaglandin E2 levels (204). In a related study, equine bone marrow-derived MSCs overexpressing IL-10 were able to provide anti-inflammatory effects in a stimulated, co-culture OA model. However, this did not translate to a protective effect on the extracellular matrix (ECM) or a rescue of ECM loss in the transduced co-cultures (90). Further study in an equine model demonstrated that an AAV5 vector overexpressing IL-10 provided rapid and sustained IL-10 expression following direct intra-articular delivery. Importantly, IL-10 levels could be detected in plasma, synovial fluid, and synovial membrane of treated joints until day 84 compared to PBS injected controls without any adverse synovial response (205). These studies demonstrate the feasibility of delivering IL-10 into diseased joints and provide support for further *in vivo* investigations into the chondroprotective effects of IL-10 for OA therapy.

Proteoglycan 4 (PRG4), a secreted protein has also been explored as a potent chondroprotective factor in blocking the pathogenesis of osteoarthritis (116, 206). PRG4 or lubricin, produced by superficial zone chondrocytes and the synovial lining cells, is an important component of synovial fluid. It provides synovial fluid with the ability to disperse strain energy under biomechanical loading thus contributing to the lubrication and protection of articular ccartilage surfaces. Recombinant PRG4 has been reported to protect against progression of OA in rodent models (207–209). Intra-articular expression of PRG4 using a helper-dependant adenoviral vector has been reported to provide protection against the development of both age-related and post traumatic OA in a mouse model (210). Further, transcriptional profiling studies revealed that PRG4 overexpression inhibits the transcriptional networks associated with cartilage catabolism and hypertrophy through the up-regulation of hypoxia inducible factor 3α (HIF3α), thus protecting against cartilage degradation and development of OA. Bone morphogenetic proteins (BMPs) are a class of proteins that have generated interest in recent years for their role in musculoskeletal repair. Recombinant protein injections of BMP2 and BMP7 has shown improved cartilage healing in previous studies (211, 212). BMP7 has been shown to enhance cartilage matrix synthesis and chondrogenic ability of chondrocytes modified by an adenovirus vector encoding BMP-7 in a bovine *ex vivo* model (213) and an equine model (70). However, more extensive studies are needed to identify and establish these additional therapeutic targets for their potential role in OA therapy.

## Future directions and conclusions

This review summarizes the current state of the field of gene therapy with emphasis on preclinical studies using the horse as an experimental model for human OA. We outline the recent advancements in viral vector-based delivery systems and potential therapeutic targets for OA therapy. The field of gene therapy has weathered many setbacks, however, technological advancements resulting in the development of safe and effective vectors and delivery methods have paved the way for increased acceptance and renewed interest in the field. There have been several successful clinical trials in human medicine (Luxterna^®^ for congenital retinal degeneration and Zolgensma^®^ for spinal muscular atrophy) which resulted in positive effects on the quality of life of the patients. The preclinical studies outlined in this review demonstrate the feasibility and relevance of using the equine joint as a translational model to explore treatment strategies for OA using gene therapy. However, high manufacturing costs associated with vector production and the inherent expensive nature of equine research pose significant challenges to undertaking large preclinical studies using this model. Improvements in vector development technology would likely lead to decreased production costs in the future, however, a growth in resources available for equine research would be vital in moving the field forward.

## Author contributions

PT and LRG were responsible for drafting the review and revising it critically. PT, JNP, JCG, RJS, CWM, and LRG were involved in revisions and approval of the final version. All authors have read and approved the final submitted manuscript.

## Funding

This work was supported by internal funding from the Orthopaedic Research Center.

## Conflict of interest

The authors declare that the research was conducted in the absence of any commercial or financial relationships that could be construed as a potential conflict of interest.

## Publisher's note

All claims expressed in this article are solely those of the authors and do not necessarily represent those of their affiliated organizations, or those of the publisher, the editors and the reviewers. Any product that may be evaluated in this article, or claim that may be made by its manufacturer, is not guaranteed or endorsed by the publisher.
