# Corrigendum: Gene therapy approaches for equine osteoarthritis

**DOI:** 10.3389/fvets.2022.1117776

**Published:** 2023-01-04

**Authors:** Parvathy Thampi, R. Jude Samulski, Joshua C. Grieger, Jennifer N. Phillips, C. Wayne McIlwraith, Laurie R. Goodrich

**Affiliations:** ^1^Orthopaedic Research Center, C. Wayne McIlwraith Translational Medicine Institute, College of Veterinary Medicine, Colorado State University, Fort Collins, CO, United States; ^2^Gene Therapy Center, University of North Carolina, Chapel Hill, NC, United States

**Keywords:** osteoarthritis, gene therapy, cartilage, adeno-associated viral vectors, translational

In the published article, there was an error in affiliation 1. Instead of “C. Wayne McIlwraith Translational Research Institute,” it should be “C. Wayne McIlwraith Translational Medicine Institute.”

The authors apologize for this error and state that this does not change the scientific conclusions of the article in any way. The original article has been updated.

